# A lower cost method of preparing corn stover for *Irpex lacteus* treatment by ensiling with lactic acid bacteria

**DOI:** 10.5713/ajas.19.0344

**Published:** 2019-10-21

**Authors:** Sasa Zuo, Di Jiang, Dongze Niu, Mingli Zheng, Ya Tao, Chuncheng Xu

**Affiliations:** 1 College of Engineering, China Agricultural University, Beijing 100083, China

**Keywords:** Ensiling, *Irpex Lacteus*, Corn Stover, Sterilization, *In vitro* Gas Production

## Abstract

**Objective:**

This study investigated a method of preparing corn stover for *Irpex lacteus* (*I. lacteus*) treatment to improve its *in vitro* rumen degradability under non-sterile conditions.

**Methods:**

Corn stover was inoculated with *Lactobacillus plantarum* (*L. plantarum*), *Lactobacillus buchneri* (*L. buchneri*), and an equal mixture of these strains, and ensiled for 0, 3, 7, 14, and 28 days. After each period, a portion of the silage was sampled to assess the silage quality, and another portion of the silage was further treated with *I. lacteus* at 28°C for 28 d. All the samples were analyzed for fermentation quality, chemical composition, and *in vitro* gas production (IVGP) as a measure of rumen fermentation capacity.

**Results:**

Lactic acid bacteria (LAB) was found to improve the silage quality of the corn stover, and the corn stover silage inoculated with *L. plantarum* produced more lactic acid and higher IVGP than other silage groups. The *I. lacteus* colonies flourished in the early stage of corn stover silage, especially on the 3-d corn stover silage inoculated with both *L. plantarum* and *L. buchneri*. This led to an 18% decrease in the acid detergent lignin content, and a 49.6% increase in IVGP compared with the raw stover.

**Conclusion:**

The combination of ensiling with the mixed LAB inoculation and *I. lacteus* treatment provided a cost-effective method for the improvement of the IVGP of corn stover from 164.8 mL/g organic matter (OM) to 246.6 mL/g OM.

## INTRODUCTION

Lignocellulosic biomass such as corn stover is an important substrate for ruminant feed. However, because lignocellulosic feeds contain recalcitrant lignin, which is intertwined with hemicellulose and cellulose, such feeds cannot be optimally used by ruminants. Therefore, effective treatment to decrease the recalcitrance of the corn stover is essential. Biological treatment using white-rot fungi is viable and safe alternative to other treatments [1]. However, the white-rot fungal treatment of biomass has been considered difficult to carry out on a large scale because of the costly process of sterilization before the fungal treatment [2].

To overcome the disadvantage of high-cost autoclave-based sterilization before the fungal treatment of the substrates, a few studies have been devoted to developing a biological treatment method that works under non-sterile conditions. Vasco-Correa et al [3] increased the inoculum ratio to 30% dry basis, which yielded a successful fungal treatment of non-sterile miscanthus by *Ceriporiopsis subvermispora*; however, it is difficult to domesticate the fungus, and a large amount of inoculum is required. Song et al [4] developed a novel method by disinfecting the stover in a 0.1% (w/v) hydrated lime solution for 24 h at room temperature before fungal treatment of corn stover, however, a lot of water is wasted in the process of washing the alkaline corn stover until the pH is neutral, and the resulting alkaline solute is an environmental pollutant. Another method is to wash the corn stover after ensiling before applying the white-rot fungal treatment to increase biogas production [5] and enzymatic hydrolysis [6]. Although this method is effective and efficient, the washing step wastes both time and water.

Ensiling is a method commonly applied on farms to preserve animal feed for off-season use. During this process, epiphytic lactic acid bacteria (LAB) transforms soluble carbohydrates into lactic acid, thereby reducing the pH to around 4.0. The naturally occurring acidic conditions can inhibit the growth of undesirable microorganisms [7], and such conditions can also partially hydrolyze hemicellulose and cellulose [8]. The disadvantage of ensiling as a treatment is that it can only make small improvements in digestibility compared to other treatments. In addition, if the initial number of epiphytic LAB is low, the pH of the forages cannot be reduced rapidly [7], and the ensiled substrate is then susceptible to aerobic aeration, which causes loss of dry matter (DM) and appearance of toxic substances [9]. Therefore, the use of LAB inoculants is recommended. In recent years, homofermentative LAB species such as *Lactobacillus plantarum* (*L. plantarum*) and heterofermentative LAB such as *Lactobacillus brevis* are the main inoculants used in silage. Their different fermentation properties exhibit various effects on the silage quality [9]. However, no studies have been published, which compare homofermentative and heterofermentative LAB, and a mixture of them on the effect of the corn stover silage quality.

The use of white-rot fungi as an additional treatment shows promise, especially some white-rot fungi that can prevail under acidic conditions [10]. Therefore, our hypothesis is that employing the acidophilic white rot fungal treatment of the ensilage corn stover could improve its *in vitro* rumen degradability without high cost sterilization. *Irpex lacteus* (*I. lacteus*), a kind of medicinal fungus, allows for efficient enzymatic conversion of the plant material and can forcefully degrade the acid detergent lignin (ADL) [11,12]. In the present study, *I. lacteus* was employed to treatment of corn stover silages inoculated with LAB to further increase its nutritional potential. The changes in the fermentation quality, numbers of microorganisms, and chemical composition during ensiling and after *I. lacteus* treatment, were analyzed to assess the effect of different LAB on the silage quality and the following fungal degradation characteristics. The *in vitro* gas production (IVGP) values of the intact, ensiled and fungal-treated-ensiled stover were compared to evaluate nutritional value of the stover.

## MATERIALS AND METHODS

### Raw material

Corn stover of the Grain and Forage corn variety was collected in October 2017 from farmland in Hebei Province, China. The stubble height of the corn stover was 20 cm and the above-ground plant including the stalks, leaves, rachis, and tassels. This material was initially air-dried at room temperature for one month. The stover was chopped to average sizes of 2 cm length, and then sealed in plastic bags and stored at room temperature for further use.

### Ensiling

The moisture content of the prepared corn stover was adjusted to 70%. After thorough mixing, it was separated into four groups, and each group received one of the following treatments: i) sterilized distilled water (CK), ii) *L. plantarum* (GenBank accession number: SUB3928584) at 1×10^5^ colony forming units (CFU)/g of fresh matter (LP), iii) *Lactobacillus buchneri* (*L. buchneri*) strain (GenBank accession number: KY828224) at 1×10^5^ CFU/g of fresh matter (LB), and iv) cells of *L. plantarum* and *L. buchneri* were mixed in an equal ratio at a concentration of 1×10^5^ CFU/g of fresh matter (MIX). The various inocula were scattered in each group and mixed thoroughly by hands with sterile gloves were worn at room temperature, approximately two hundred grams of the pre-ensiled material was packed into a plastic film bag silo (Hiryu KN type, 180×260 mm; Asahikasei, Tokyo, Japan), and this was followed by air removal using a vacuum sealer (BH 950; Matsushita, Tokyo, Japan). Silos were prepared in triplicate and stored at ambient temperature for 0, 3, 7, 14, and 28 d.

### White-rot fungus of *Irpex lacteus* pre-culture and treatment

The fungus of *I. lacteus* (CGMCC-5.809) was obtained from the China General Microbiological Culture Collection Center (Beijing, China), and was preserved on potato dextrose agar (PDA) slants at 4°C. Initial cultures were developed on PDA plates at 28°C until mycelium covered the entire agar plates. The spawn was prepared by adding 20 pieces (approx. 1 g) of colonized agar culture (0.2 cm^2^) to 200 g of sterilized 70% moisture content of wheat grains. The inoculated wheat grains were incubated at 28°C until all grains were colonized by mycelium. The spawn was stored at 4°C until further use. After unsealing at 0, 3, 7, 14, and 28 d, 100 g of silage was weighed into 25×17 cm polyethylene bags (Miaojie, Wuxi, Jiangsu, China). Thereafter, 4 g of the pre-cultured wheat grains with *I. lacteus* were inoculated in 0, 3, 7, 14, and 28 d of ensiled corn stover. All bags were incubated at 28°C for 28 d in a controlled humidity chamber at 85% humidity. Triplicate samples of each silage group treated with *I. lacteus* were set as: CKI, LPI, LBI, and MIXI. The corn stover silages and the silages after treated with *I. lacteus* were analyzed for the changes in the quality of fermentation, chemical composition, and IVGP.

### Fermentation quality and microbial enumeration

To measure fermentation quality, 10 g of silage, with or without *I. lacteus* treatment was homogenized with 90 mL of sterilized distilled water and submerged for 3 h in the 4°C fridge, and then filtered through qualitative filter paper. The filtrates were used for determining pH and organic acid. The pH was measured using a glass electrode pH meter (S20K, Mettler Toledo, Greifensee, Switzerland), and the organic acid of lactic, acetic, propionic and butyric acid content was determined by HPLC (column: Shodex RS Pak KC-811, Showa Denko K.K., Kawasaki, Japan; detector: DAD, 210 nm, SPD-20A, Shimadzu Co., Ltd, Kyoto, Japan; eluent: 3 mmol/L HClO4, 1.0 mL/min; temperature: 50°C) using the procedures described by Xu et al [13].

Populations of microorganisms were measured through the spread-plate count method. The LAB were counted on Man Rogosa Sharpe agar (Difco Laboratories, Detroit, MI, USA) incubated at 37°C for 48 h under anaerobic conditions. Aerobic bacteria were counted on nutrient agar (Nissui-Seiyaku Ltd., Japan) incubated at 30°C for 48 h under aerobic conditions. Molds and yeasts were counted on PDA (Nissui-Seiyaku Ltd., Tokyo, Japan) plates incubated at 28°C for 72 h under aerobic conditions. The colonies were counted from the plates at appropriate dilutions, and the number was expressed as CFU per gram of fresh matter.

### Chemical analysis

The DM content was determined by drying the materials in an oven at 65°C for 48 h, and water soluble carbohydrates (WSC) content was determined using the method of Owens et al [14]. Fiber analysis was carried on air-dried, ground (1 mm) material according to Van Soest et al [15], using an Ankom fiber analyser 2000 (ANKOM Technology, Macedon, New York, USA). Neutral detergent fiber (aNDF) was determined with heat stable amylase without adding sodium sulfite. The aNDF, acid detergent fiber (ADF), ADL were expressed inclusive of residual ash. Neutral detergent solute (NDS) content was calculated by subtracting the aNDF from 1,000. The hemicellulose content was calculated as the difference between aNDF and ADF contents and the cellulose content was calculated as the difference between ADF and ADL contents. Absolute numbers were calculated according to Van Kuijk et al [16] using data on the remaining constant weight of air-forced oven DM of the corn stover silage, with or without fungal treatment, and were expressed as gram and representative average values between the duplicates.

### *In vitro* gas production technique

The IVGP was measured according to Menke and Steingass [17]. In summary, rumen fluid of fistulated Angus bullocks fed a corn silage, and wheat straw-based diet was mixed with a buffer solution under continuous flushing with CO_2_. Air dried samples (220 mg) were incubated in 30 mL buffered rumen fluid for 72 h at 39°C. The IVGP of all the samples was manually recorded at 0, 2, 4, 6, 8, 10, 12, 16, 20, 24, 30, 36, 42, 48, 60, and 72 h of incubation, and related to the organic matter (OM) content. The data shown here represent average values between the triplicates.

### Statistical analysis

Data on fermentation quality, microbial counts, chemical compositions, with and without *I. lacteus* inoculation, were subjected to two-way analysis of variance analysis of variance with the fixed effects of silage pretreatments and storage periods using the generalized linear model (GLM) procedures of SPSS, version 21.0 (IBM Corp., Armonk, NY, USA). This was followed by Duncan’s multiple range tests and significance was defined at a 0.05 probability level. The 72 h IVGP before and after a 28 d fungal treatment compared with the silages uninoculated with LAB was subjected to GLM analysis in SAS 9.2 with the following model:

$$Y_{ij}=\mu +\alpha_{i}+\omega_{ij}$$

in which *Y_ij_* is the observation *j* for pretreatment *i*; *μ* is the overall mean; *α_i_* is the fixed effect of pretreatment *i*; *ω_ij_* is the random error.

## RESULTS AND DISCUSSION

### Fermentation properties and microbial components of corn stover silage before and after *I. lacteus* treatment

The first part of the experiments was performed as part of a broad research effort aimed at determining the effect of different LAB inoculants on corn stover silage quality and which method of inoculation can be better provide a good non-sterile environment for white rot fungi further treatment of the stover. The changes in the pH and organic acid content of the corn stover during ensiling are shown in Table 1. The group of MIX has the lowest pH, while the CK has the highest pH (p<0.01), and the pH decreased quickly before day 7 and then stabilized. The lactic acid content in all the pretreatment samples increased at the initial stage of the silage, reached the highest (p<0.01) value on day 7, and then decreased thereafter. These results are demonstrated that high LAB counts are needed to ensure a rapid and vigorous fermentation that results in a rapid decline in pH and accumulation of the lactic acid. In this study, the LP group had the highest (p<0.01) lactic acid content, for the LAB fermentation proceeded rapidly with *L. plantarum*, which resulted in the intensive and fast production of lactic acid and a rapid decrease in pH, indicating that homofermentative LAB can significantly improve natural silage fermentation quality [18]. However, the homofermentative LAB inoculants can impair the aerobic stability of silage, while the present result with a high acetic acid content in the LB group probably can improve aerobic stability of silage, for the heterofermentative lactic acid bacterium, such as *L. buchneri*, has been studied as an additive to improve the aerobic stability of silage by producing high levels of acetic acid in silage [19]. However, in this study, an even higher acetic acid content in MIX group indicates a stronger suppression potential of undesirable microorganisms inoculated with a mixture of LAB strains. Also, the MIX group had the total highest (p<0.01) propionic acid content, followed by the LB and then the LP. The CK had the lowest (p<0.01) acetic acid and propionic acid content. Moreover, the butyric acid content in the CK was significantly higher than that of the other three silage groups (p<0.01), indicating that the CK silage has probably undergone clostridial fermentation; therefore, the CK presented poor silage quality.

The fermentation characteristics after *I. lacteus* treatment are shown in Table 2. The pH of the CKI was highest (p<0.01), which was probably due to the poor silage quality and bad aerobic stability of the CK, and thus it was easily contaminated by mold. The period of ensilage had no significant effect on the pH of each group’s samples after the 28-d fungal treatment; however, the shorter the ensilage period with the LAB inoculation, the more the *I. lacteus* thrived and the lower pH after the fungal treatment (p = 0.023) which is most likely due to the initial pH of the silage and the amount of organic acid suitable for the fungus to germinate. As the silage period prolonged, the accumulation of organic acid or other components might have limited the growth of *I. lacteus* [5,6]. The lactic acid content of the silage samples decreased after the 28 d of the *I. lacteus* treatment, and the 3-d MIXI showed the highest lactic acid content (p<0.01). Acetic acid was only detected in the CKI and LPI. The propionic acid content of the CKI was higher than it was in the other groups (p<0.01). Butyric acid was not detected in any of the *I. lacteus* treatment samples. The organic acids decreased after the *I. lacteus* treatment, which was probably due to the aerobic microbes metabolizing lactic acid and butyric acid for their growth [20].

**Figure 1:**
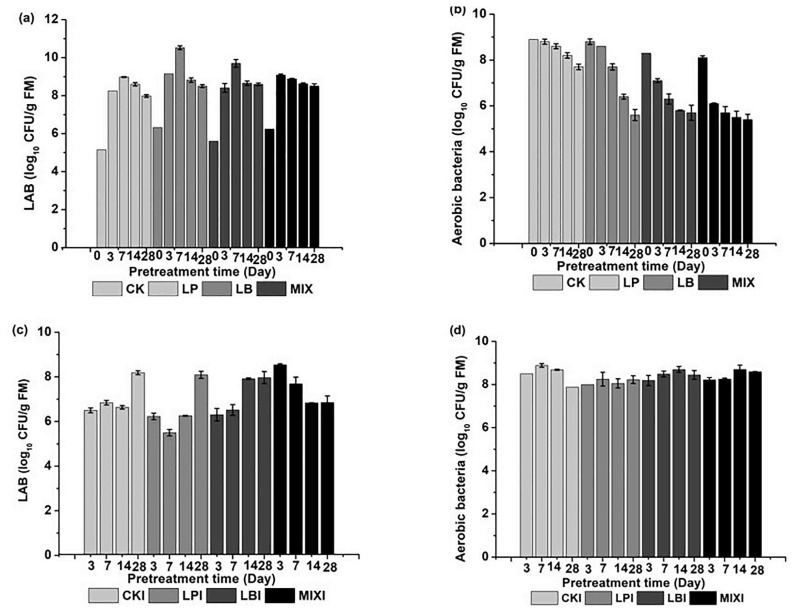
The numbers of LAB (a), aerobic bacteria (b) at 0, 3, 7, 14, 28 d of corn stover ensiling, and the LAB (c), aerobic bacteria (d) numbers after the *Irpex lacteus* treatment of different periods of the silage. LAB, lactic acid bacteria; CK, silage uninoculated with LAB; LP, silage inoculated with *Lactobacillus planturam*; LB, silage inoculated with *Lactobacillus buchneri*; MIX, silage inoculated with the mixed *L. planturam* and *L. buchneri*; CKI, *I. lacteus* treatment of corn stover silage uninoculated with LAB; LPI, *I. lacteus* treatment of silage inoculated with *Lactobacillus plantarum*; LBI, *I. lacteus* treatment of silage inoculated with *Lactobacillus buchneri*; MIXI, *I. lacteus* treatment of silage inoculated with mixed *L. plantarum* and *L. buchneri*. Data are presented as means of three replicates (mean±standard deviation).

Silage that has been inoculated with LAB is commonly fed to ruminants because the LAB reproduction accumulates lactic acid, which is the key factor for ensuring good forge quality (both enhanced storage capacity and improved digestibility) during ensiling [21]. The initial epiphytic LAB and enterobacteria in corn stover were 1.4×10^5^ and 5×10^8^ CFU/g fresh matter respectively. During ensiling, LAB as well as the aerobic bacteria number was determined (Figure 1a, b). In this study, the homofermentative LAB fermentation proceeded rapidly by adding *L. plantarum*, which produced the highest numbers of the LAB (Figure 1a). However, the peak value of LAB numbers appearing on the 3-d MIX group and the peak at day 7 for the other three groups indicate that MIX can enter the silage stable period more quickly than other groups. The aerobic bacteria number was observed to decrease during ensiling of corn stover; an especially rapid decrease was observed at the beginning of the MIX group (p<0.01) (Figure 1b). Moreover, the aerobic bacteria number in the inoculated LAB silages was lower than in the CK group during the process (p<0.01; Figure 1b). Indeed, by the end of the MIX process, the number of aerobic bacteria had decreased to 3×10^5^ CFU/g fresh matter (Figure 1b), which indicates a significantly inhibitory effect of mixed LAB on the growth of aerobic bacteria. The decrease in the numbers of aerobic bacteria, yeast and mold (data not shown) after ensiling, was attributed to the stress from the anaerobic environment, organic acids and low pH [22]. Therefore, for development of functional bacterial inoculants, both homofermentative and heterofermentative LAB should be involved.

Aerobic stability of silage is a key factor in ensuring that silage provides well-preserved nutrients to animals without contamination of mold spores and toxins. As the corn stover silage without LAB inoculation (0-d CK) has the poor aerobic stability, which was completely contaminated by mold a few days after treatment with *I. lacteus*, no analysis of microorganism numbers, fermentation quality, chemical composition, and gas production of the *I. lacteus* treatment of the 0-d silages was done. The numbers of LAB decreased, and the number of aerobic bacteria increased overall after *I. lacteus* treatment of the different days of silage compared with the stover before the fungal treatment (Figure 1c and d), because when the silage bags were opened, acid-resistant aerobic bacteria began to multiply growth under aerobic condition, which suppressed the LAB growth. The 7-d LPI had the lowest LAB numbers (3.25×10^5^ CFU/g fresh matter) (p<0.01; Figure 1c). However, the 3-d MIXI had the highest LAB numbers (3.53× 10^8^ CFU/g fresh matter) (p<0.05; Figure 1c), which was most likely due to the higher aerobic stability of this time frame and this treatment. The numbers of aerobic bacteria in all of the groups was above 1×10^8^ CFU/g fresh matter after the *I. lacteus* treatment, and there is no difference of the number between the different ensilage periods combined with *I. lacteus* treatment (Figure 1d). Although some of the multiply growth of acid-resistant microorganisms potentially inhibited the growth of white-rot fungi, it has been reported that *I. lacteus* has a stronger ability to compete with these bacteria, which might be the reason why *I. lacteus* can grow well in the ensiled corn stover that contain high amounts of aerobic bacteria [23]. Moreover, the high abundance of *I. lacteus* in the MIXI and the low abundance of this fungus in the CKI indicated that the inoculation of LAB provided a good environment for the growth of *I. lacteus*, which made *I. lacteus* became the dominant fungus in the corn stover silage (Supporting information).

### Chemical analysis of corn stover silage before and after *I. lacteus* treatment

The aNDF, ADF, ADL, NDS, and WSC content of the corn stover used in the silage fermentation is 742.9±5.9, 462.6±4.2, 86.9±1.6, 257.1±5.9, and 19.8±1.3, respectively. For all silage groups, the absolute weight of DM, cellulose, hemicellulose, ADL, NDS, and WSC decreased with the greater ensilage period (Table 3). The efficient conservation of corn stover silage with minimal DM losses during the storage period is important to provide ruminants with essential nutrients. In this study, the DM losses in corn stover silage during ensiling were small and within the acceptable range of 10%. The absolute weight of the DM of the CK was the lowest after 28-d ensilage period (p<0.01), and the DM weight for LP and LB was equivalent but was higher than the DM weight of MIX (p = 0.024). The amount of cellulose in the LP was lower than the amount in the LB, but higher than MIX (p<0.01). No difference was found in the weight of hemicellulose or ADL between the different silage groups. The loss of cellulose and hemicellulose observed in corn stover silage during ensiling is consistent with studies conducted by Yahaya et al [24], which suggests that cellulose and hemicellulose could serve as microbial substrates for the production of acids, thus contributing to the higher acids production. The absolute amount of NDS in the LP and MIX was higher (p< 0.01) than LB, while the CK had the lowest amount of NDS (p<0.01). The WSC is the mainly component of NDS. It was reported that WSC is the best source of fermentable material for the production of high quality silages by LAB [25]. For all the groups, the amount of WSC decreased with the greater ensilage time, and more than half of WSC absolute amount was lost after 28 d of ensilage. The weight of WSC in the LB was lowest (p<0.01) because CO_2_ is formed during the conversion of lactic acid to acetic acid and 1,2-propanediol in the heterofermentative fermentation process, which leads to greater WSC loss [26]. While the LP retained a relatively high WSC content after 28 d of ensilage, these results are in accordance with the acid production during ensiling.

After the 28-d *I. lacteus* treatment, the absolute amount of DM was calculated based on the data presented in the Table 3 and further decreased (Table 4). However, the rate of degradation of DM, cellulose, hemicellulose and ADL after the *I. lacteus* treatment slowed with prolonged ensilage because the lowering of pH probably inhibited the growth of *I. lacteus*. The DM and cellulose amount of the CK after *I. lacteus* treatment was lower than the amount in the other groups of the same period (p<0.01), which is probably because the aerobic bacterial consumed more of the substrates in the CKI. A lower hemicellulose and ADL weight was found in 3 d, 7 d, and 14 d LPI (p<0.01), and the change of hemicellulose is accompanied with the loss of the ADL. Interestingly, the lowest (p<0.01) ADL amount and the highest amount of NDS and WSC were observed in the 3-d MIXI (p<0.01), which is consistent with the speed of *I. lacteus* growth.

### *In vitro* gas production of corn stover silage before and after *I. lacteus* treatment

**Figure 2:**
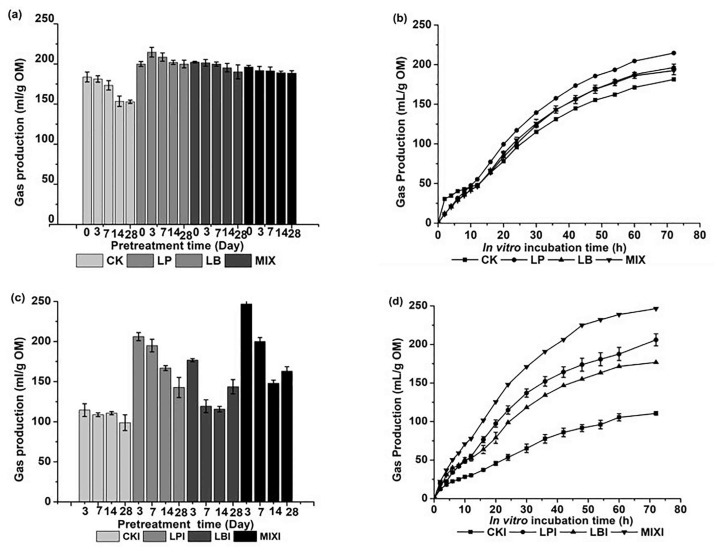
*In vitro* gas production (IVGP) of different periods of corn stover silage (a), and the IVGP dynamic changes of 3 d different silage groups (b); as well as the IVGP of *Irpex lacteus* treatment of the different periods of silage (c), and the IVGP dynamic changes of the *I. lacteus* treatment of the 3 d different silage (d). CK, silage uninoculated with LAB; LP, the silage inoculated with *Lactobacillus planturam*; LB, the silage inoculated with *Lactobacillus buchneri*; MIX, the silage inoculated with the mixed *L. planturam* and *L. buchneri*; CKI, *I. lacteus* treatment of corn stover silage uninoculated with LAB; LPI, *I. lacteus* treatment of silage inoculated with *Lactobacillus plantarum*; LBI, *I. lacteus* treatment of silage inoculated with *Lactobacillus buchneri*; MIXI, *I. lacteus* treatment of silage inoculated with mixed *L. plantarum* and *L. buchneri*. Data are presented as means of three replicates (mean±standard deviation).

The silage inoculated with LAB presented higher IVGP than the CK (p<0.01; Figure 2a). With prolonged ensilage, the IVGP gradually decreased (Figure 2a). The IVGP of samples that spent different time’s in silage indicates that the more time in silage, the less accessible nutrients utilized by the rumen microorganisms, which is consistent with the changes of DM, cellulose, hemicellulose, NDS, and WSC content during ensiling. The 3-d LP showed a higher IVGP than other 3-d silage groups (p<0.01), and a 20.5% increase compared with the raw stover. The increased IVGP in 3-d LP is probably due to *L. plantarum* decreasing the pH, and raising the lactate:acetate ratio [27]. Also, the improved the silage quality, which preserved more true protein during silage fermentation than was present in the CK, which in turn increased in vitro ruminal microbial growth [28]. In addition, although heterofermentation of *L. buchneri* can improve the aerobic stability of the corn silage, it will cause substrate nutrient loss due to carbon dioxide gas production. That is the reason why IVGP is higher in LP than LB. In addition, the gas production can be divided into two or three phases which were caused by the fermentation of the soluble, insoluble but degradable, and undegradable proportions. Soluble components have the highest contribution to the gas production during the first phase and the insoluble components mainly contribute to the gas production of the second phase [29]. The IVGP rate of the CK was higher than other groups during the first 12 h (Figure 2b), suggesting a greater soluble portion in the 3-d CK corn stover silage. However, the 72 h total IVGP of the CK was lower than that of the other three groups. After 12 h, not only did the IVGP rate of 3-d LP silage increased, but also the volume of the gas was higher than other 3-d silage groups (p<0.01; Figure 2b). The total IVGP of the silage samples indicated that the LAB can improve the silage quality of the corn stover, especially the silage inoculated with *L. plantarum*.

For the silage treated with *I. lacteus*, the 3-d silage with *I. lacteus* treatment showed a higher IVGP than other periods of silages treated with the fungus (Figure 2c). At 3 d of ensiling, the pH and amount of organic acids of the samples are probably more appropriate for the *I. lacteus* growth, which allows it to more effectively degrade the ADL; therefore, the cellulose was more accessible to the rumen microorganisms. With prolonged ensilage, the accumulation of organic acid might inhibit the fungus growth [5]. The highest (p<0.01) IVGP was observed in the 3-d MIXI, which reached 246.6 mL/g OM, significantly higher than the IVGP of the 3-d CKI, which was only 114.5 mL/g OM (p<0.01; Figure 2c), and also higher than the IVGP of the raw corn stover, which was 164.8 mL/g OM (p<0.01) (data not shown). The IVGP of the 3-d and 7-d LPI was higher than the 3-d LBI (p<0.01; Figure 2c). Although *L. buchneri* can improve the aerobic stability of the silage so that it is better for the *I. lacteus* growth, the fungus growth is even greater in the silage samples inoculated with *L. plantarum* and the lactic acid, NDS, and WSC content of the 3-d LPI is higher than the LBI. The ADL content of the LPI is lower than that of LBI, therefore, which may explain the higher IVGP after *I. lacteus* treatment of LP than LB. The IVGP rate of the 3-d MIXI was highest (p<0.01; Figure 2d). This is because it had the lowest content of ADL and the highest content of NDS and WSC. The increased amount of IVGP was not different from that of our previous research’s result of an IVGP of 249.74 mL/g OM after the same days of *I. lacteus* treatment of the sterilized corn stover [30], indicating that the present method is effective and efficient. The IVGP rate of the 3-d CKI was lowest (p<0.01; Figure 2d), lower than even the raw stover (p<0.01). This is probably due to the poor quality of the silage which was easily contaminated by the aerobic bacteria, which inhibited the growth of the *I. lacteus*. Moreover, contaminating microorganisms also consumed a portion of nutritious portion of the samples during the fungal treatment.

## CONCLUSION

This study sought to evaluate an alternative method to prepare corn stover for *I. lacteus* treatment to optimize its nutritional value. The IVGP was improved after 28 d of *I. lacteus* treatment of the 3-d corn stover silage pre-treated with the mixed of *L. plantarum* and *L. buchneri*. Further work is needed to evaluate the effect of the combination of ensiling with fungus-treatment on *in situ* degradation and ruminal fermentation. Overall, the combination of *I. lacteus* treatment for a short period of silage inoculated with a mixture of LAB is a promising technology alternative to high cost sterilization before the fungal treatment.

## Figures and Tables

**Table 1:** Changes in pH and organic acids (g/kg dry matter) content of corn stover during the 28 d of ensilage

Items	Treatments	Days of silage					SEM	p-value1)		
	
		0 d	3 d	7 d	14 d	28 d		T	D	T×D
pH	CK	7.06^Aa^	5.87^Ab^	4.98^Ac^	5.47^Ad^	5.70^Ae^	0.115	<0.001	<0.001	<0.001
	LP	6.87^Ba^	4.70^Bbc^	4.48^Cc^	4.64^Bbc^	5.06^Bb^				
	LB	6.88^Ba^	5.26^Cb^	4.76^Bc^	4.65^Bc^	4.65^Cc^				
	MIX	6.77^Ca^	4.61^Dbc^	4.42^Cc^	4.64^Bb^	4.64^Cbc^				
LA	CK	0.59^Bc^	7.94^Db^	28.30^Ba^	8.55^Db^	0.11^Cc^	1.875	<0.001	<0.001	<0.001
	LP	2.46^Ac^	19.48^Bb^	35.42^Aa^	34.12^Aa^	16.69^Ac^				
	LB	0.62^Bc^	13.68^Cb^	25.94^Ba^	25.18^Ba^	14.23^Bb^				
	MIX	2.1^Ad^	24.76^Aa^	27.7^Ba^	19.63^Cc^	15.73^Bb^				
AA	CK	2.93^Bc^	3.50^Dc^	5.09^Db^	5.70^Db^	8.27^Da^	1.219	<0.001	<0.001	<0.001
	LP	2.39^Ce^	8.69^Cd^	11.10^Cc^	12.14^Cb^	12.77^Ca^				
	LB	2.99^Be^	11.48^Bd^	14.75^Bc^	18.22^Bb^	25.91^Ba^				
	MIX	4.00^Ae^	14.39^Ad^	16.51^Ac^	23.56^Ab^	29.10^Aa^				
PA	CK	3.54^d^	4.76^Bd^	6.65^Bc^	10.83^ABb^	13.29^Ba^	1.013	<0.001	<0.001	<0.001
	LP	3.00^d^	4.44^Bd^	6.49^Bc^	9.81^Bb^	14.98^Ba^				
	LB	3.47^b^	3.80^Bb^	5.18^Bb^	15.82^Aa^	19.54^Aa^				
	MIX	3.95^d^	9.45^Ac^	11.24^Ac^	14.00^ABb^	19.88^Aa^				
BA	CK	0.315^d^	3.50^Ac^	9.41^Ab^	16.45^Aa^	18.46^a^	1.165	<0.001	<0.001	<0.001
	LP	0.29^b^	0.36^Bb^	0.41^Bb^	2.61^Ba^	0.33^b^				
	LB	0.42^b^	0.57^Bab^	0.66^Ba^	0.11^Bc^	ND2)				
	MIX	0.40^ab^	0.68^Ba^	0.42^Bb^	0.29^Bb^	ND				

SEM, standard error of the mean; CK, silage uninoculated with lactic acid bacteria; LP, silage inoculated with *Lactobacillus plantarum*; LB, silage inoculated with *Lactobacillus buchneri*; MIX, silage inoculated with mixed *L. plantarum* and *L. buchneri*; LA, lactic acid; AA, acetic acid; PA, propionic acid; BA, butyric acid.

1) T, effect of different pretreatment; D, effect of silage time; T×D, interaction between pretreatment and silage time.

2) ND, not detected.

Means in the same row (^a–e^) or in the same column (^A–D^) with different superscripts differ (p<0.05).

**Table 2:** pH and organic acids (g/kg dry matter) content after *Irpex lacteus* treatment of 3, 7, 14, and 28 d of corn stover silage

Items	Treatments	Days of silage				SEM	p-value1)		
	
		3 d	7 d	14 d	28 d		T	D	T×D
pH	CKI	6.76^Aa^	6.10^Ab^	5.61^Ac^	5.62^Ac^	0.104	<0.001	0.142	<0.001
	LPI	4.73^Bc^	4.79^Bbc^	4.84^Bb^	5.03^ABa^				
	LBI	4.79^B^	4.84^B^	5.02^B^	4.94^AB^				
	MIXI	4.63^Cb^	4.94^Ba^	4.79^Bab^	4.73^Bb^				
LA	CKI	2.30^Ca^	1.42^Bb^	1.52^Bb^	0.91^Cc^	0.195	<0.001	<0.001	<0.001
	LPI	3.62^ABa^	3.03^Ab^	2.08^Ac^	1.09^Cd^				
	LBI	3.00^BCa^	0.63^Cd^	1.06^Cc^	1.65^Ab^				
	MIXI	4.0^Aa^	0.74^Cd^	2.55^Bb^	1.33^Bc^				
AA	CKI	ND2)	ND	ND	0.25	-	-	-	-
	LPI	ND	ND	ND	0.43				
	LBI	ND	ND	ND	ND				
	MIXI	ND	ND	ND	ND				
PA	CKI	1.58	1.47	0.85	0.35	-	-	-	-
	LPI	ND	ND	0.05	0.01				
	LBI	ND	1.38	0.30	0.11				
	MIXI	ND	0.94	0.45	0.30				
BA	CKI	ND	ND	ND	ND	-	-	-	-
	LPI	ND	ND	ND	ND				
	LBI	ND	ND	ND	ND				
	MIXI	ND	ND	ND	ND				

SEM, standard error of the mean; CKI, *I. lacteus* treatment of corn stover silage uninoculated with LAB; LPI, *I. lacteus* treatment of silage inoculated with *Lactobacillus plantarum*; LBI, *I. lacteus* treatment of silage inoculated with *Lactobacillus buchneri*; MIXI, *I. lacteus* treatment of silage inoculated with mixed *L. plantarum* and *L. buchneri*; LA, lactic acid; AA, acetic acid; PA, propionic acid; BA, butyric acid.

1) T, effect of *I. lacteus* treatment of different silges; D, effect of silage time; T×D, interaction between treatment and silage time.

2) ND, not detected.

Means in the same row (^a–d^) or in the same column (^A–C^) with different superscripts differ (p<0.05).

**Table 3:** Changes of chemical composition (g) of corn stover during the 28 d of ensilage

Items	Pretreatments	Days of silage					SEM	p-value1)		
	
		0 d	3 d	7 d	14 d	28 d		T	D	T×D
DM	CK	1,000^a^	970^Bb^	938^Cc^	920^Cd^	918^Cd^	3.93	<0.001	<0.001	<0.001
	LP	1,000^a^	983^Ab^	980^Ab^	971^Ac^	945^Bd^				
	LB	1,000^a^	984^Ab^	977^Ab^	967^Ac^	963^Ac^				
	MIX	1,000^a^	982^Ab^	962^Bc^	957^Bc^	956^Bc^				
Cell	CK	378^a^	373^Aa^	360^Bb^	350^Bc^	346^Bc^	1.52	<0.001	<0.001	<0.001
	LP	378^a^	360^Bb^	357^Bb^	357^Bb^	356^Bb^				
	LB	378	378^A^	369^A^	372^A^	375^A^				
	MIX	378^a^	347^Cc^	355^Bb^	354^Bb^	347^Bc^				
HC	CK	266^a^	247^b^	241^b^	239^b^	237^b^	1.63	0.075	<0.001	0.899
	LP	266^a^	254^b^	246^b^	249^b^	246^b^				
	LB	266^a^	261^a^	250^b^	244^bc^	242^c^				
	MIX	266^a^	255^b^	242^bc^	237^c^	232^c^				
ADL	CK	86.9	85.3	81.1	79.8	78.0	0.64	0.125	<0.001	0.357
	LP	86.9^a^	74.6^b^	79.0^b^	76.6^b^	75.5^b^				
	LB	86.9^a^	76.1^b^	76.6^b^	77.7^b^	76.7^b^				
	MIX	86.9	80.2	78.7	80.3	80.5				
NDS	CK	265	260^B^	258^B^	254^C^	253^B^	1.98	<0.001	<0.001	<0.001
	LP	275^b^	297^Aa^	296^Aa^	283^Aa^	268^Ab^				
	LB	270^b^	273^Bb^	283^Aa^	271^Bb^	265^Ac^				
	MIX	273^bc^	298^Aa^	286^Ab^	285^Ab^	266^Ac^				
WSC	CK	19.8^a^	8.1^Ab^	7.2^Abc^	6.9^Abc^	6.3^Ac^	0.74	<0.001	<0.001	<0.001
	LP	22.1^a^	8.7^Ab^	8.4^Ab^	8.0^Ab^	7.6^Ab^				
	LB	20.4^a^	6.7^Bb^	2.2^Bc^	1.1^Bcd^	0.4^Cd^				
	MIX	20.8^a^	4.1^Cb^	2.0^Bc^	2.6^Bc^	2.5^Bc^				

SEM, standard error of the mean; DM, dry matter; CK, silage uninoculated with LAB; LP, silage inoculated with *Lactobacillus plantarum*; LB, silage inoculated with *Lactobacillus buchneri*; MIX, silage inoculated with mixed *L. plantarum* and *L. buchneri*; Cell, cellulose; HC, hemicellulose; ADL, acid detergent lignin; NDS, neutral detergent solute; WSC, Water soluble carbohydrate.

1) T, effect of different pretreatment; D, effect of silage time; T×D, interaction between pretreatment and time.

Means in the same row (^a–d^) or in the same column (^A–C^) with different superscripts differ (p<0.05).

**Table 4:** Chemical composition (g) of corn stover after *Irpex lacteus* treatment of 3, 7, 14, and 28 d of ensilage.

Items	Treatments	Days of silage				SEM	p-value1)		
	
		3 d	7 d	14 d	28 d		T	D	T×D
DM	CKI	793^Cd^	814^Cc^	824^Cb^	840^Ca^	3.70	<0.001	<0.001	<0.001
	LPI	828^Bb^	831^Bb^	870^Aa^	872^Aa^				
	LBI	822^Bb^	828^Bb^	829^Cb^	850^Ba^				
	MIXI	840^Ac^	841^Ac^	847^Bb^	869^Aa^				
Cell	CKI	265^Bb^	271^Ba^	274^Ca^	277^Ca^	2.40	<0.001	<0.001	<0.001
	LPI	277^ABc^	288^Ab^	306^Aa^	308^Aa^				
	LBI	272^ABb^	279^ABab^	286^BCab^	293^Ba^				
	MIXI	285^Aa^	283^ABa^	291^Ba^	274^Cb^				
HC	CKI	168^b^	171^Ab^	179^Ab^	198^a^	2.42	<0.001	<0.001	<0.351
	LPI	145^b^	151^Bb^	164^Bab^	174^a^				
	LBI	164	164^AB^	179^A^	172				
	MIXI	151^b^	161^ABab^	169^ABab^	173^a^				
ADL	CKI	89.3^Ac^	90.1^c^	95.6^Ab^	102^a^	1.85	0.006	<0.001	0.002
	LPI	77.3^Bb^	79.2^b^	85.8^Bb^	106^a^				
	LBI	79.7^Bc^	86.1^b^	86.5^Bb^	98.7^a^				
	MIXI	71.2^Cb^	96.7^a^	99.2^Aa^	104^a^				
NDS	CKI	270^C^	283^C^	275^B^	260^C^	3.87	<0.001	<0.001	<0.001
	LPI	328^Aa^	320^Aab^	314^Ab^	284^Bc^				
	LBI	306^Ba^	299^BCab^	277^Bc^	286^Bbc^				
	MIXI	333^Aa^	307^ABb^	288^ABc^	318^Ab^				
WSC	CKI	12.7^Ba^	10.4^b^	11.9^ABab^	10.6^Bb^	0.54	<0.001	<0.001	0.001
	LPI	13.7^Ba^	13.5^a^	13.2^Aa^	12.2^Bb^				
	LBI	12.9^Ba^	11.1^b^	9.1^Bc^	13.5^Ba^				
	MIXI	21.1^Aa^	11.7^c^	12.3^ABc^	18.3^Ab^				

SEM, standard error of the mean; DM, dry matter; CKI, *I. lacteus* treatment of corn stover silage uninoculated with LAB; LPI, *I. lacteus* treatment of silage inoculated with *Lactobacillus plantarum*; LBI, *I. lacteus* treatment of silage inoculated with *Lactobacillus buchneri*; MIXI, *I. lacteus* treatment of silage inoculated with mixed LAB; Cell, cellulose; HC, hemicellulose; ADL, acid detergent lignin; NDS, neutral detergent solute; WSC, water soluble carbohydrate.

1) T, effect of *I. lacteus* treatment of different silges; D, effect of silage time; T×D, interaction between treatment and silage time.

Means in the same row (^a–d^) or in the same column (^A–C^) with different superscripts differ (p<0.05).
